# Does *Toxoplasma gondii* Infection Affect the Levels of IgE and Cytokines (IL-5, IL-6, IL-10, IL-12, and TNF-alpha)?

**DOI:** 10.1155/2009/374696

**Published:** 2009-05-25

**Authors:** Joanna Matowicka-Karna, Violetta Dymicka-Piekarska, Halina Kemona

**Affiliations:** Department of Clinical Laboratory Diagnostics, Medical University of Bialystok, J. Waszyngton'a 15A, 15-269 Bialystok, Poland

## Abstract

In the study performed in a group of patients infected with *T. gondii*, we evaluated Th2 humoral response (IL-5, IL-6, IL-10) and Th1 cell response (IL-12, TNF-*α*). The study objective was to assess the effect of *T. gondii* on chosen indices of the immune response. The study involved 52 women infected with *T. gondii* (aged 18–42 years) prior to antiparasitic treatment. In all the patients, we found IgM (index >0.7) and IgG which exceeded 300 IU/ml. The control group (C) consisted of 40 healthy women aged 18–46 years. In the study group (T) and in the control group (C), the levels of IgE, IL-5, IL-6, IL-10, IL-12, and TNF-*α* were determined. In our study, *T. gondii* patients had twofold higher levels of IL-5 and IL-6 as compared to healthy subjects, which seems to confirm the presence of an inflammatory state. We found the level of IL-10 to be fivefold higher in the course of *toxoplasmosis* than in healthy controls. The levels of IL-12 and TNF-*α* were comparable to those observed in healthy controls. The study has revealed that patients infected with *T. gondii* show increased production of the humoral response cytokines, whereas the generation of the cell response cytokines remains unchanged.

## 1. Introduction

*Toxoplasma gondii* is a ruthless intracellular parasite belonging to *Coccidae*. *T. gondii* occurs in three forms: tachyzoites, bradyzoites (in tissues), and sporozoites. The parasite locates in the brain, heart, lungs, and most frequently in the lymph nodes. *Toxoplasmosis* is a disease affecting 500 million people worldwide. The seroprevalence varies (from 5% to 90%), depending on geographical location, age, habit of eating raw meat or unwashed fruit and vegetables, and general level of hygiene. The incidence of infections is higher in warmer and humid climate and increases with age. The disease can be congenital or acquired [1–4]. The parasite after invading the human body multiplies inside the cell, causing damage to the reticuloendothelial system. Rapid multiplication of the parasite and formation of the so-called pseudocysts are characteristics of the acute phase of invasion [5]. 

In parasitic invasions, an increase is observed in the production of IgE antibodies, especially in helminth infections. This defect results from disturbances in the regulation of antibody production by Th cells, which promotes local inflammatory reaction. Via release of mediators from mast cells IgE participates in the reaction of antibody-dependent cellular cytotoxicity (ADCC). Cytotoxic activity of eosinophils is increased under the influence of cytokines (TNF-*α* and IL-5) released by mast cells, lymphocytes, and macrophages [6]. Th2 lymphocytes synthesize specific cytokines (IL-4, IL-5, IL-6, IL-10, IL-13, and IL-14), which play a major role in the pathogenesis of parasitic diseases. IL-5 is the major cytokine responsible for the increase in the eosinophil population in parasitoses, whereas IL-6 stimulates production of antibodies and exerts a proinflammatory effect by stimulating the generation of acute phase proteins. IL-10 and IL-12 control the type of the immune response. The former inhibits cytokine synthesis and by blocking the production of IL-6 and TNF-*α* causes an advantage of the response occurring with Th2 involvement and B cell activation. However, IL-12 facilitates formation of a Th1 type response [7–10].

In the current study, performed on a group of patients infected with *T. gondii, *we evaluated humoral response (IL-5, IL-6, IL-10 secreted by Th2 cells) and cell response (IL-12 and TNF-*α* secreted by Th1 cells). The study objective was to assess the effect of *T. gondii* on chosen indices of the immune response. 

## 2. Materials and Methods

The study involved 52 women infected with *T. gondii* (aged 18–42 years) prior to antiparasitic treatment. All these women had swollen cervical and nuchal lymph nodes (toxoplasmic lymphadenopathy), although other diseases were excluded (mononucleosis, cytomegaly, granulomatosis, lymphomas, leukemias, AIDS). Tests were performed in the women who were not able to conceive. In all the patients (group T), the levels of specific IgM and IgG antibodies, directed against *T. gondii,* were determined. In all the women, IgM was present (index > 0.7) and the level of IgG exceeded 300 IU/ml. The diagnosis of the early phase of *T. gondii* infection was based on the detection of a significant increase in the levels of specific IgG antibodies of low avidity, with the co-occurrence of IgM antibodies. Assays were performed using ELFA on an immunoserological analyzer VIDAS with a set of reagents (bioMerieux). The control group (C) consisted of 40 healthy women aged 18–46 years, clinically asymptomatic, who underwent prophylactic examinations and had no anti-*T. gondii* specific antibodies.

The levels of IgE, IL-5, IL-6, IL-10, IL-12, and TNF-*α* were determined in the study group (T) and in the control group (C). 

### 2.1. Determination of Total IgE

Total IgE level was determined in 100 *μ*l serum samples by ELFA (bioMerieux, France) on an immunoserological analyzer VIDAS. The sensitivity threshold of the method is 0.5 KIU/l. 

### 2.2. Determination of the Levels of Interleukins (IL-5, IL-6, IL- 10, IL-12) and TNF-*α*

The levels of interleukins and TNF-*α* were determined in blood serum using ELISA method with a set of Quantikine human (R&D, USA). 

The results were subjected to statistical analysis using the program Statistica 8.0. The differences were considered statistically significant when the value of test function was at the level of significance set at *P* < .05. The Kolgomorov consistency test was used for features consistent with normal distribution, the *t*-Student test for comparisons between the groups and the Mann-Whitney test for traits inconsistent with this distribution. 

## 3. Results

 In patients infected with *T. gondii* the mean IgE level was 74.7 ± 105.5 IU/l, being twice as high as the mean IgE value in healthy individuals (37.7 ± 27.6 IU/l). The difference between these values is statistically significant (*P* < .05).

The mean level of IL-5 in *T. gondii* infection was 7.02 ± 2.9 pg/ml. However, in healthy subjects, the IL-5 concentration was markedly lower (mean 3.5 ± 1.6 pg/ml). The difference between these values is statistically significant (*P* < .0001). 

The mean level of IL-6 in *T. gondii-*infected patients was 5.21 ± 3.5 pg/ml, being twice as high as the mean value in healthy controls (2.45 ± 1.4 pg/ml). The difference in the values of IL-6 between the infected and healthy subjects is statistically significant (*P* < .0001). 

The level of IL-10 was found to be most highly differentiated. In the course of *toxoplasmosis* its mean level was 4.50 ± 2.9 pg/ml whereas in healthy controls only 0.76 ± 0.2 pg/ml. The difference between these values is highly statistically significant (*P* < .0001). However, a comparison of the mean levels of IL-12 and TNF-*α* between *T. gondii* patients and healthy subjects did not reveal any statistically significant differences (0.4 < *P* < .5).

## 4. Discussion

 The prevalence of *T. gondii* has been reported to increase with age (>40 years) and in women consuming raw meat, vegetables, and fruit [11]. In the acute phase, directly after invading the body, *T. gondii* begins to multiply rapidly. In a majority of cases, acquired *toxoplasmosis* is asymptomatic. In the second week of infection, specific IgM antibodies are present in the blood. IgE antibodies also appear at the same time, slightly preceding specific IgA antibodies, but they are not found 4 months after the infection onset [12]. In our study, the concentration of IgE antibodies in patients infected with *T. gondii* was statistically significantly higher than in healthy subjects, which was accompanied by an elevated level of IgM antibodies, thus suggesting an acute form of invasion. Increased IgE level correlates with early acute inflammation or with a reactive form of *toxoplasmosis*. However, negative IgE test result does not exclude acute *toxoplasmosis* [13]. Dziubek et al. [14] who determined specific antibodies directed against *T. gondii* (IgM, IgG, IgA) and CD4+ and CD8+ lymphocyte subpopulations found more substantial involvement of CD8+, whereas lower expression of CD4+ lymphocytes might be due to a suppressive action of the parasite.

The humoral response involves IL-5 and IL-6 that exert a proinflammatory effect and IL-10 inhibiting the action of IL-6. Patients infected with *T. gondii* had twofold higher values of IL-5 as compared to healthy subjects. IL-5 prolongs eosinophil survival, stimulates eosinophil degranulation and production of reactive oxygen species, and exerts a chemotactic effect on these cells [15]. It has been proved that *Toxocara canis* invasion promotes release of IL-5, IL-8, and TNF-*α*, which is accompanied by eosinophilia with an inflammatory state [16]. However, Nickdel et al. [17] have shown that at the early stage of *T. gondii* infection, coexisting with eosinophilia and increased level of IL-5, pathological changes are prominent in the small intestine, which is accompanied by a simultaneous reduction in IL-12 and IFN-*γ*. We also noted an increase in the level of IL-5 and a decrease in the level of IL-12. However, Filisetti and Candolfi [18] revealed an IL-5 induced increase in IL-12 production in *toxoplasmosis*. A decrease in the production of IL-12 and inhibition of Th1 response have been reported by Lang et al. [7].

In our study, *T. gondii* patients had a twofold higher level of IL-6 as compared to healthy subjects, which seems to confirm the presence of an inflammatory state. The major function of IL-6 is the involvement in the immune response through the action on lymphocytes B. It is a mediator responsible for the production of acute phase proteins and increased cytotoxic activity of NK cells. IL-6 is an early and sensitive, although nonspecific, marker of inflammatory states.

We found the level of IL-10 to be fivefold higher in the course of *toxoplasmosis* than in healthy controls. Therefore, we could have expected a decrease in IL-6 production, which was however not found. IL-10 plays an essential role in the inflammatory response during acute *T. gondii* infection, since it inhibits the cellular-type immune response (IL-12, TNF-*α*) and inflammatory response (IL-6) [17, 19]. IL-10 counteracts the harmful effects of the inflammatory response which is based on the increased production of TNF-*α*, IFN-*γ*, and NO associated with intestinal multiplication of *T. gondii* [20]. IL-10 is able to deactivate macrophages, induce IFN-*γ* by *T. gondii*, and facilitate intracellular parasite survival. IL-10 induces immunosuppression during *T. gondii* invasion, which is beneficial both for the host and the parasite [7].

Also the cellular response, involved among others IL-12 and TNF-*α*, plays an important role in parasitic invasions. In our patients infected with *T. gondii*, the levels of IL-12 and TNF-*α* were comparable to those observed in healthy controls. IL-12 was found to increase the production of IgG but not to inhibit IgE. IFN-*γ*, IL-2, and IL-12 are involved in the protection against parasitic invasions. IL-12 stimulates the production of IFN-*γ* and TNF-*α* and activates lymphocyte cytotoxicity [1]. Involvement of IFN-*γ* and IL-10 in the immune mechanisms directed against *T. gondii* has been described by Kang et al. [21]. According to Filisetti and Candolfi [18], secretion of IFN-*γ* in mice increases the phagocytic activity of macrophages and cytotoxic activity of CD8+T lymphocytes. Increased production of IFN-*γ* is strongly correlated with parasite virulence and enhanced apoptosis.

In our study, in the course of *toxoplasmosis* the levels of TNF-*α* and IL-12 did not change, whereas the concentration of IL-5 increased. However, as reported by Lang et al. (2007) *T. gondii* inhibits production of TNF-*α* and IL-12. TNF-*α* is a cytokine of inflammatory and immune response and together with IL-6 can enhance proliferation and differentiation of B lymphocytes [7]. TNF-*α* activates eosinophil cytotoxicity toward protozoa and induces secretion of acute phase proteins via IL-6 production. TNF-*α* and IFN-*γ* have antiproliferative properties. In *toxoplasmosis,* TNF-*α* appears to be indispensable for macrophage activation and inhibition of parasite replication; however, this is possible only in cooperation with IFN-*γ*. 

The study has revealed that patients infected with *T. gondii* show increased production of the cytokines responsible for humoral response, whereas generation of the cell response cytokines remains unchanged. 
